# The Phenotype of Circulating Follicular-Helper T Cells in Patients with Rheumatoid Arthritis Defines CD200 as a Potential Therapeutic Target

**DOI:** 10.1155/2012/948218

**Published:** 2012-10-04

**Authors:** Aron Chakera, Sophia C. Bennett, Olivier Morteau, Paul Bowness, Raashid A. Luqmani, Richard J. Cornall

**Affiliations:** ^1^School of Medicine and Pharmacology, Sir Charles Gairdner Hospital, The University of Western Australia, 4th Floor G Block, Hospital Avenue, Nedlands, Perth, WA 6009, Australia; ^2^Nuffield Department of Clinical Medicine, University of Oxford, Old Road Campus, Roosevelt Drive, Henry Wellcome Building for Molecular Physiology, Oxford OX3 7BN, UK; ^3^Nuffield Department of Rheumatology, Orthopaedics and Musculoskeletal Science, Nuffield Orthopaedic Centre, Windmill Road, Oxford OX3 7HE, UK

## Abstract

Rheumatoid arthritis (RA) is a systemic autoimmune disease primarily affecting synovial joints in which the development of autoantibodies represents a failure of normal tolerance mechanisms, suggesting a role for follicular helper T cells (T_FH_) in the genesis of autoimmunity. To determine whether quantitative or qualitative abnormalities in the circulating T_FH_ cell population exist, we analysed by flow cytometry the number and profile of these cells in 35 patients with RA and 15 matched controls. Results were correlated with patient characteristics, including the presence of autoantibodies, disease activity, and treatment with biologic agents. Circulating T_FH_ cells from patients with RA show significantly increased expression of the immunoglobulin superfamily receptor CD200, with highest levels seen in seropositive patients (*P* = 0.0045) and patients treated with anti-TNF**α** agents (*P* = 0.0008). This occurs in the absence of any change in T_FH_ numbers or overt bias towards Th1, Th2, or Th17 phenotypes. CD200 levels did not correlate with DAS28 scores (*P* = 0.887). Although the number of circulating T_FH_ cells is not altered in the blood of patients with RA, the T_FH_ cells have a distinct phenotype. These differences associate T_FH_ cells with the pathogenesis of RA and support the relevance of the CD200/CD200R signalling pathway as a potential therapeutic target.

## 1. Introduction

Rheumatoid arthritis (RA) is a chronic, systemic autoimmune disease characterised by inflammation of synovial joints [1]. The aetiology of RA is both complex and poorly understood, and while the formation of autoantibodies such as rheumatoid factors (RFs) or anticitrullinated protein antibodies (ACPAs) is common [2, 3], their role in disease pathogenesis remains unclear. Autoantibodies in RA are usually of the IgG subclass and demonstrate high affinity for their targets, characteristics consistent with production by B-cells that have undergone T-cell-dependent germinal centre (GC) maturation [4]. A role for CD4^+^ T cells in disease development is further supported by their presence in the synovium of affected patients, often with evidence of ectopic germinal centre formation RA [5] and the association of RA with HLA-DR4 [6, 7]. 

Within germinal centres, the fate of developing B cells is determined by their ability to present antigen to a specialised subset of CD4^+^ T cells termed follicular helper T cells (T_FH_), located in the B-cell follicle by virtue of expression of the chemokine receptor CXCR5 [8]. Through a combination of cytokine secretion and highly regulated cell-cell interactions, T_FH_ cells guide the maturation of B cells, facilitating class-switching and somatic hypermutation [9]. T_FH_ cells also provide critical censoring functions, withdrawing help from B cells with autoreactive potential thereby preventing autoimmunity [10, 11]. A central role for T_FH_ cells in the development of autoimmune diseases has been confirmed in animal models, where dysregulated T_FH_ function can promote autoantibody formation [12, 13], and in humans, with increased T_FH_ cell numbers identified in some patients with SLE and RA and an altered T_FH_ phenotype demonstrable in patients with juvenile dermatomyositis [14–17]. 

One of the difficulties of systematically studying T_FH_ cells in human autoimmune conditions is that historically T_FH_ cells were defined not only by the receptors they express but also by their anatomical location: secondary lymphoid organs, making routine analysis of these cells impractical [8, 18]. However, recently circulating populations of T-helper cells that express CXCR5 and have similar functionality to tissue-resident T_FH_ cells (provision of B-cell help, expression of the transcription factor Bcl6 and the cytokine IL-21) have been defined [16, 17]. Analysis of these cells therefore provides an opportunity to interrogate the T_FH_ compartment through sampling of peripheral blood.

To determine whether T_FH_ cells might be relevant to the pathogenesis of RA, we examined whether quantitative or qualitative abnormalities exist in the circulating T_FH_ population in patients with RA and whether these differences might be more pronounced in seropositive patients (the presence of class-switched autoantibodies being indicative of T_FH_ cell-induced maturation). In contrast to previous work, we did not find increased numbers of circulating T_FH_ cells in patients with RA; however the phenotypic profile of these cells was abnormal, with increased expression of the inhibitory receptor CD200. Improved understanding of the spatial and temporal regulation of stimulatory and inhibitory receptors present on T_FH_ cells may provide new insights into the development of autoimmunity in RA.

## 2. Materials and Methods

### 2.1. Patient Recruitment and Clinical Samples

Patients attending rheumatology and orthopaedic clinics were recruited to donate whole blood following written informed consent. Healthy controls were recruited through advertisement and donated blood following written informed consent. Research was conducted in accordance with the Declaration of Helsinki. Ethical approval for the study was granted by the Berkshire Research Ethics Committee (REC reference 08/H0607/50). A total of 50 subjects were recruited (35 patients with RA and 15 controls). All patients fulfilled the American Rheumatological Association's criteria for the diagnosis of RA [19], and disease activity scores (DAS28-CRP) were recorded for each patient at the time of recruitment. Patients with RA were further subdivided for analysis based on the presence of circulating autoantibodies and treatment with anti-TNF*α* agents. 20 patients were autoantibody positive; 19 had rheumatoid factor, 8 had ACPA, and 7 patients were positive for both. Characteristics of the study population are shown in Table 1. 

### 2.2. Reagents

Directly conjugated anti-human antibodies against CD3, CD4, CXCR5, CD45RO, CD69, CD95, CD134 (OX-40), ICOS, CD200, CD150, CXCR3, CCR6, and HLA-DR were purchased from BD Biosciences (San Jose, California). 

### 2.3. Phenotypic Analysis of T_**FH**_ Cells from Whole Blood

3 mL of whole blood was washed twice with 10 mL PBS (Fisher Scientific) before centrifugation at 400 g for 5 minutes. Following aspiration of the PBS/plasma, 100 *μ*L aliquots were transferred into polystyrene FACS tubes (BD Biosciences) for staining. Directly conjugated surface antibodies were added and cells incubated at 4°C for 30 minutes in the dark. Red blood cells were lysed with 2 mL of BD Lyse/Fix (BD Biosciences) for 10 minutes at 37°C. Samples were then washed in 3 mL of 2% BSA (Fisher Scientific)/PBS and resuspended in 150 *μ*L of 2% Paraformaldehyde/PBS before acquisition on a BD FACSCanto flow cytometer. 

### 2.4. Serum Exchange Experiments

Serum was aspirated from clotted blood following centrifugation at 1500 g before being snap frozen on dry ice and stored at −80°C. For serum exchange experiments, serum from two seropositive patients with high T_FH_ cell CD200 levels and two seropositive patients with negligible T_FH_ CD200 levels was thawed and added to fresh PBMCs from healthy controls (without significant CD200 expression on their T_FH_ cells). Cells were cultured (1 × 10^6^/well) in RPMI media containing 5% patient serum, 5% human serum (Sigma-Aldrich, Dorset, UK), or 5% human serum supplemented with PHA (5 *μ*g/mL).

### 2.5. Data Analysis

Flow cytometry was performed on a BD FACSCanto machine using BD FACSDiva software. Flow cytometry data were analysed using FlowJo 8.8.3 (Tree Star, Inc., Ashland, OR). Statistical analyses were performed with Prism 5 software (GraphPad Software, Inc. La Jolla, CA). Normality was assessed with the D'Agostino and Pearson test. Nonparametric data were analysed using the Mann-Whitney or Kruskal-Wallis tests. Significance is shown as * = *P* < 0.05, ** = *P* < 0.01, *** = *P* < 0.001.

## 3. Results/Figures

### 3.1. Follicular Helper T-Cell Numbers Are Not Increased in Patients with RA

**Figure 1:**
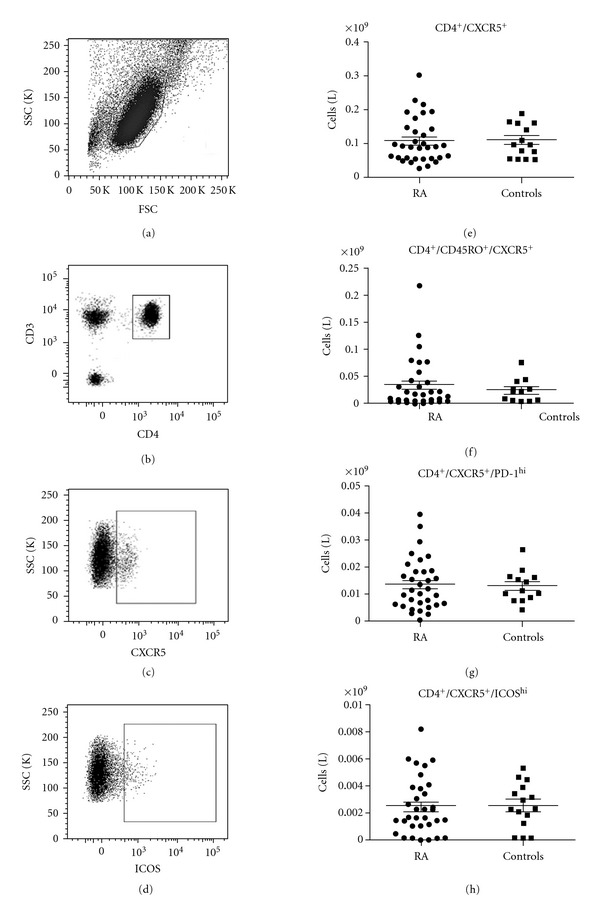
Enumeration of circulating T_FH_ cells in whole blood. T_FH_ cells were counted in whole blood using four different phenotypic definitions. (a)–(d) Representative gating strategy. (e) CD4^+^/CXCR5^+^; (f) CD4^+^/CD45RO^+^/CXCR5^+^; (g) CD4^+^/CXCR5^+^/PD-1^hi^; (h) CD4^+^/CXCR5^+^/ICOS^hi^. No differences were detected between patients with RA and controls (*P* ≥ 0.4 in all cases). Spots represent individual patients. Mean ± SEM are shown.

Data from animal studies and from patients with SLE or juvenile dermatomyositis have suggested important roles for T_FH_ cells in the development of autoimmunity. As ectopic germinal centres are common in the synovium of patients with RA and a previous study in treatment-naïve patients with RA demonstrated increased numbers of circulating T_FH_ cells, we sought to confirm whether increased numbers of circulating T_FH_ cells are present in patients with established disease, potentially contributing to the breakdown in tolerance. To answer this question, we analysed T_FH_ numbers in whole blood from 35 patients with active RA (average age 59, range 33–90) and 15 controls (average age 56, range 33–85) (*P* = 0.50). Detailed characteristics of the study population are shown in Table 1. As the most appropriate way to define T_FH_ cells in peripheral blood continues to be debated, we calculated T_FH_ cell numbers using four different phenotypic definitions: (i) CD4^+^/CXCR5^+^, (ii) CD4^+^/CD45RO^+^/CXCR5^+^, (iii) CD4^+^/CXCR5^+^/PD-1^hi^, and (iv) CD4^+^/CXCR5^+^/ICOS^hi^ (Figure 1), as absolute cell counts (per mL of whole blood) and as a % of total CD4^+^ cells (data not shown). There was no difference in T_FH_ cell numbers between controls or patients with RA (including the subgroup of seropositive patients) regardless of the definition used (*P* ≥ 0.4 in all cases).

### 3.2. T_**FH**_ Subsets Are Not Polarized toward Th1, Th2, or Th17 Phenotypes in RA

**Figure 2:**
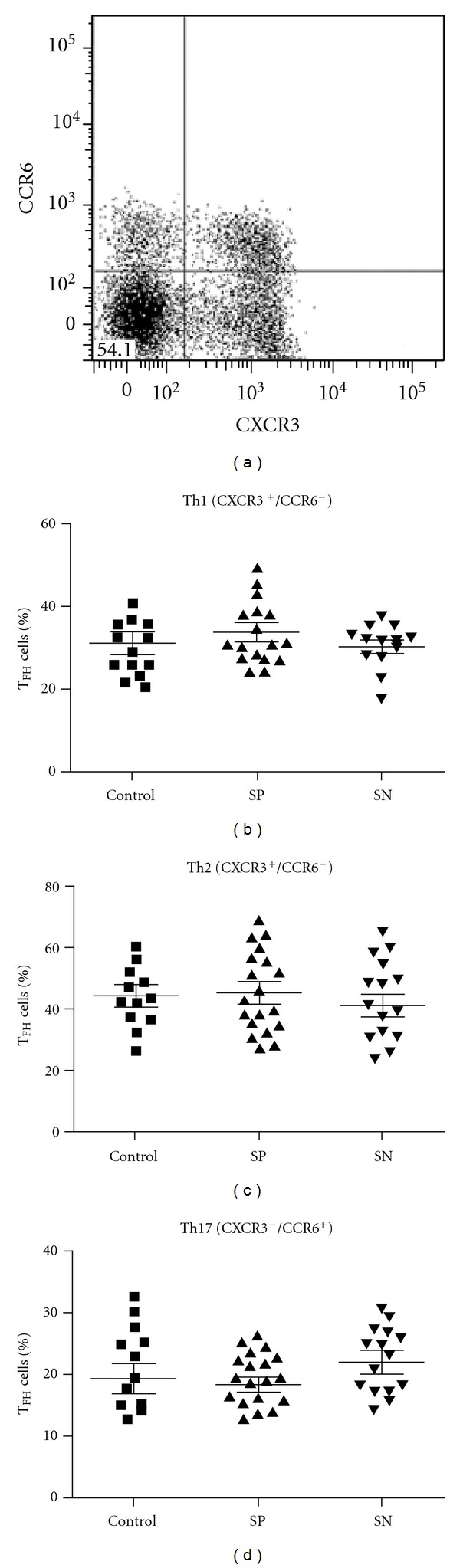
T_FH_ cell subsets in RA. The % of T_FH_ cells with Th1, Th2, or Th17 phenotypes was assessed through expression of the chemokine receptors CXCR3 and CCR6 (see text for details). SP: seropositive, SN: seronegative. Spots represent individual patients. Mean ± SEM are shown.

Circulating T_FH_ cells can be further subdivided into three subsets based on the expression patterns of the inflammatory chemokine receptors CXCR3 and CCR6 [17]. These subsets display functionality associated with other T-helper cell classes (Th1, Th2, and Th17), including expression of their characteristic cytokines and transcription factors [17]. To determine whether T_FH_ subsets were biased in patients with RA, we analysed the expression of CCR6 and CXCR3 on CD4^+^/CXCR5^+^ T cells, using the definitions of Morita et al. [17]. Th1 T_FH_ cells were defined as being CXCR3^+^/CCR6^−^, Th2 T_FH_ cells as CXCR3^−^/CCR6^−^, and Th17 T_FH_ cells as CXCR3^−^/CCR6^+^. No biases toward a particular T_FH_ cell subset were detected in patients with RA or in the subgroup of RA patients with autoantibodies (*P* > 0.65 for all subsets) (Figure 2).

### 3.3. Qualitative Differences in T_**FH**_ Cells Exist in Patients with RA

**Figure 3:**
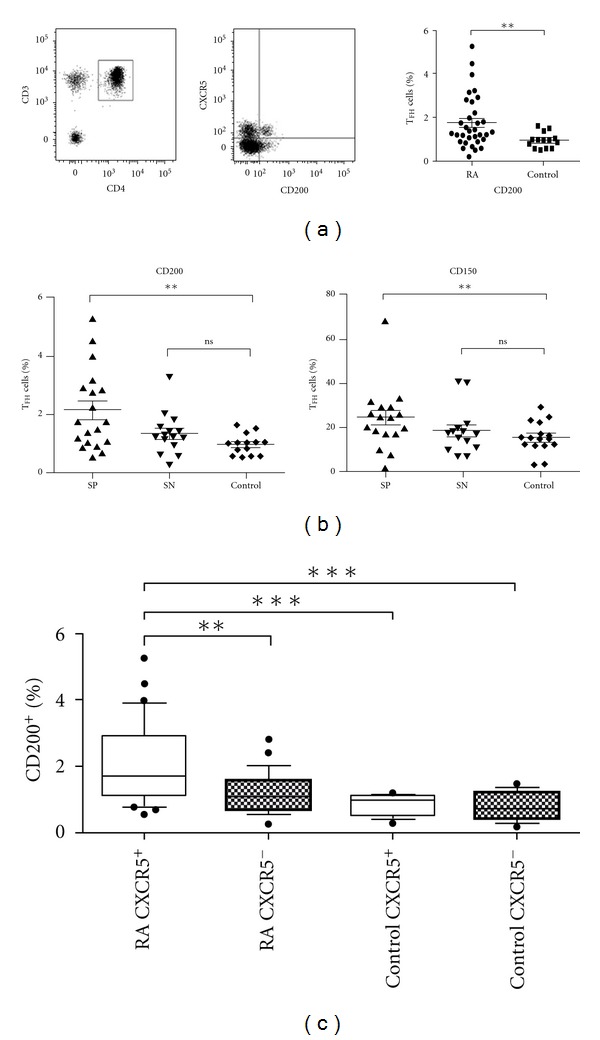
Analysis of receptors expressed by T_FH_ cells. (a) Representative gating strategy showing the increased expression of CD200 on T_FH_ cells from patients with RA. (b) Significantly increased expression of CD200 and CD150 was detected on circulating T_FH_ cells from seropositive RA patients (SP: seropositive, SN: seronegative) (*P* = 0.0045 and *P* = 0.0088, resp.). Each spot represents an individual patient, and the mean ± SEM are shown. (c) CD200 expression is enriched on CXCR5 positive CD4^+^ T cells in patients with RA.

The balance between the positive and negative signals received by a cell is critical in determining its fate. T_FH_ cells express both stimulatory and inhibitory receptors on their surface that can regulate B-cell maturation and responses to antigen [10, 20]. Therefore we asked next if qualitative differences in receptor expression might contribute to the development of autoreactive B cells in RA. To explore this possibility we reviewed the expression of receptors displayed by T_FH_ cells, including CD200, CD150, CD134 (OX-40), CD69, CD95, and HLA-DR. These assays revealed significantly increased levels of the inhibitory receptor CD200 were present in patients with RA (*P* = 0.0079) (Figure 3(a)). As the presence of autoantibodies defines a distinct subset of patients [21], we also assessed the differences in receptor expression between seropositive and seronegative patients, which demonstrated increased levels of both CD200 and CD150 on T_FH_ cells from seropositive patients with RA (*P* = 0.0045 and *P* = 0.0088 resp.) (Figure 3(b)). Although CD200 and CD150 upregulation could be detected on other CD4^+^ T cell populations, only CD200 expression was more pronounced on CXCR5^+^ cells, suggesting selective enrichment in the T_FH_ compartment (Figure 3(c)). Although there was a trend towards increased expression of the activating receptor OX40 (CD134) (see Supplementary Figure 1(a) available online at doi:1155/2012/948218), there was no enrichment in the T_FH_ compartment or in seropositive patients, and the difference was not statistically significant (*P* > 0.05). There were no differences in the expression of CD69, CD95, or HLA-DR (Supplementary Figures 1(a)–1(d)).

### 3.4. CD200 Expression Correlates with Treatment

**Figure 4:**
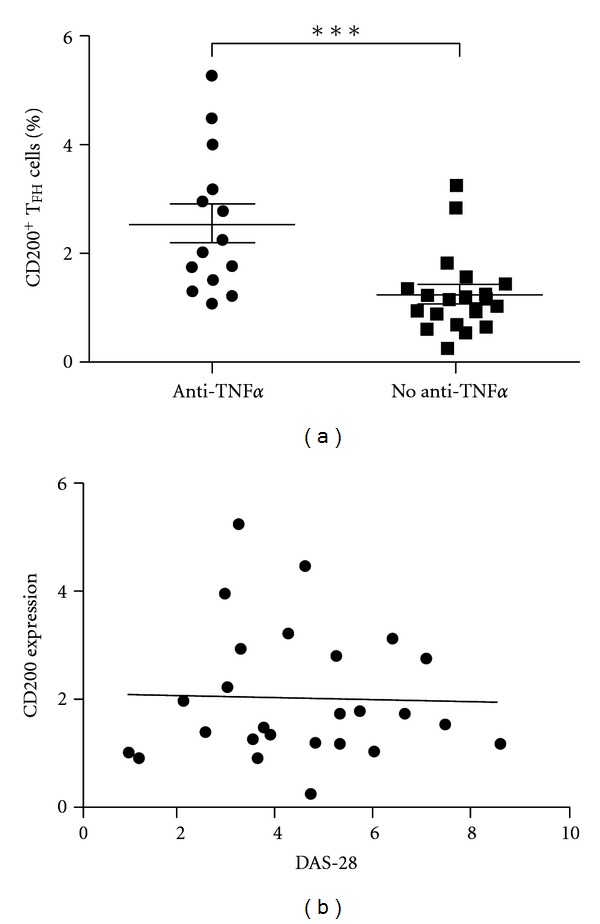
Relationship between CD200 expression on T_FH_ cells, treatment, and disease activity. (a) The % of T_FH_ cells expressing CD200 in patients being treated with anti-TNF*α* therapy was significantly increased compared with patients not on anti-TNF*α* therapy (*P* = 0.0008). The mean DAS of patients receiving anti-TNF*α* therapy was 4.679, compared with 4.418 in patients who were not (*P* = 0.875). Each spot represents an individual patient, and the mean ± SEM are shown. (b) Correlation between T_FH_ cell CD200 expression and DAS-28 score (*r* ^2^ = 0.0009, *P* = 0.887).

CD200 expression is induced by inflammation, with increased levels detected on T cells following stimulation with inflammatory mediators, including TNF*α* [22]. CD200 has been proposed to be part of a negative feedback loop (via CD200R), in which the induction of CD200 suppresses further cytokine release by macrophages [23]. Given the dramatic benefits that anti-TNF*α* therapies have on disease progression in RA we wondered if CD200 expression on T_FH_ cells might itself reflect treatment with these agents or disease activity. Levels of CD200 on T_FH_ cells were significantly higher in patients receiving treatment with anti-TNF*α* agents (*P* = 0.0008) (Figure 4(a)), regardless of whether they were seropositive (*P* = 0.053-data not shown). CD200 expression was not related to disease activity as calculated by the DAS-28 (CRP) score (*P* = 0.887, *r* ^2^ = 0.0009) (Figure 4(b)) or to the ACPA titre (*P* = 0.896, *r* ^2^ = 0.003 (data not shown)).

### 3.5. CD200 Expression on T_**FH**_ Cells Is Not Induced by Circulating Factors

To determine whether a soluble factor could be responsible for the increased expression of CD200, PBMCs from healthy controls were incubated with serum from two seropositive patients with RA known to have high levels of CD200 on their T_FH_ cells or two seropositive patients with minimal T_FH_ cell CD200 expression. CD200 levels were analysed at baseline and then every 24 hours until 72 hours. CD200 expression increased slightly over time under all conditions, but there was no difference in the percentage of T_FH_ cells expressing CD200 at any time point following addition of serum from high or low expressors (Supplementary Figure 2, *P* > 0.05). However, significantly increased expression of CD200 on T_FH_ cells was detected on freshly purified peripheral blood mononuclear cells PBMC (F) when compared with whole blood from the same donors (*P* = 0.004), suggesting that the manipulation of T_FH_ cells itself may alter expression of the receptor (Supplementary Figure 3). 

## 4. Discussion

This study is the first to describe an alteration in the phenotype of circulating follicular helper T cells in patients with rheumatoid arthritis. T_FH_ cells are a distinct subset of CXCR5-expressing CD4^+^ T cells that can localise to germinal centres and are thought to maintain tolerance by censoring B cells with specificity for self-antigens by failing to provide the necessary cytokine and costimulatory receptor support for their maturation [13, 24]. Dysregulation of this process has been reported in animal models and in humans with autoimmune diseases, particularly in conditions associated with autoantibodies [11, 13, 14, 16, 24, 25]. Aberrant T_FH_ cell function is also implicated in the development of autoimmune phenomena in patients with angioimmunoblastic T-cell lymphoma, a malignant proliferation of lymphocytes that possess a T_FH_ phenotype [26].

Given the common findings of autoantibodies and ectopic germinal centres in patients with RA [5, 27], we hypothesized that quantitative or qualitative abnormalities of T_FH_ cells may be central to the autoimmune process. As an accepted phenotype for defining the circulating counterparts of GC T_FH_ cells remains controversial, we measured T_FH_ cell numbers in patients with RA and controls using a series of different phenotypic definitions. In each case T_FH_ cell numbers were not increased in patients with RA. This is in contrast to the findings of Simpson et al. in patients with systemic lupus erythematosus (SLE), where a distinct subset of patients (~30%) was identified as having increased T_FH_ cell numbers, a stable phenotype that appeared unrelated to disease activity [16], and Ma et al. who found increased T_FH_ cell numbers in treatment-naïve patients with RA [15]. Although SLE and RA are both systemic autoimmune diseases in which autoantibody production is prominent, they preferentially affect different organ systems, and different genetic loci have been implicated in their pathogenesis [28, 29]. As the average duration of RA in our patient population was 51 months, whereas the patients in the study of Ma et al. were newly diagnosed, one explanation for the difference between our data and that of Ma et al. is that excess T_FH_ cells from the circulation may be recruited into sites of ectopic GC formation that develop over time, resulting in normal circulating numbers. The effects of different therapeutic regimens on T_FH_ development and trafficking may also be relevant, and future prospective studies will help answer these questions. 

Aberrant expression of costimulatory molecules can sustain the development of autoreactive cells causing autoimmunity, with blockade of these receptors a proven therapeutic strategy [30–34]. As T_FH_ cells are known to express multiple receptors that mediate their interactions with B cells [13, 24], we also investigated whether qualitative differences in T_FH_ cell stimulatory or inhibitory receptor expression were present in patients with RA. Using a panel of antibodies we identified significantly elevated levels of the inhibitory receptors CD200 and CD150 in patients with autoantibodies (Figure 3). 

CD200 (previously known as OX-2) is a member of the immunoglobulin gene superfamily of receptors that displays a restricted tissue distribution, including activated T and B cells [35]. CD200 is induced by inflammatory cytokines, including TNF*α* [22], and binds to CD200R, a nonclassical immunoglobulin family receptor found predominantly on macrophages and dendritic cells, but also identified in the lymph nodes and synovium of animals with collagen-induced arthritis [36]. Binding of CD200 to CD200R causes phosphorylation of the cytoplasmic domain of CD200R and signalling through a series of adaptor proteins and the MAPK pathway [37]. The consequence of this interaction is to dampen the inflammatory response, with ligation of CD200R a therapeutic target in collagen-induced arthritis [23]. The importance of the CD200-CD200R axis in autoimmunity has been confirmed in mice genetically engineered to lack expression of CD200 or where the CD200-CD200R interaction is blocked, and in patients with multiple sclerosis or androgenetic alopecia, where reduced CD200 expression is associated with disease [38–41]. As well as limiting the expansion of activated macrophages [42], CD200 can inhibit NK cell function, which may be important in the pathogenesis of NK-mediated bone destruction in patients with RA [43], while the expression of CD200R on B-cells suggests the potential for CD200^+^ T cells to directly regulate B-cell function [44].

Current models propose that the CD200-CD200R axis provides a link between adaptive and innate immune responses by regulating tissue-specific tolerance set points, high CD200 expression serving to increase the threshold for activation [45]. The increased expression of CD200 on T_FH_ cells in patients with RA may therefore represent a physiological response to inflammation designed to contain autoreactivity [46]. To assess whether a circulating factor or factors may be present in the serum of patients with high T_FH_ CD200 levels, that could be a potential biomarker, we performed a series of serum exchange experiments (Supplementary Figure 2). Serum from patients with high T_FH_ CD200 expression did not induce CD200 on control T_FH_ cells, suggesting that serum factors alone are insufficient for the induction of CD200.

As CD200 can be upregulated by TNF*α* [22], we assessed whether treatment with anti-TNF*α* agents was related to CD200 expression. Contrary to expectations, T_FH_ CD200 expression was significantly higher in patients with RA who were receiving anti-TNF*α* therapy (Figure 4(a)). One potential explanation for this finding is that patients whose disease is associated with higher TNF levels may be more likely to respond to anti-TNF*α* therapy and were therefore receiving these treatments at the time of recruitment. The expression of CD200 on T_FH_ cells might therefore identify patients suited to these agents, a hypothesis that could be tested prospectively. Although CD200 is upregulated during inflammation, most studies have focussed on acute inflammation [45]. Therefore, the failure of T_FH_ CD200 expression to correlate with disease activity (Figure 4(b)) may reflect the chronicity of the inflammatory process in RA or the presence of more complex interactions between treatment and systemic versus local synovial TNF*α* concentrations. 

As the trafficking of T_FH_ subsets is still poorly understood, one possibility is that T_FH_ cells with proinflammatory phenotypes are trapped within GCs, biasing the profile seen in the peripheral circulation. Although skewing of the distribution of T_FH_ subsets has been implicated in the pathogenesis of autoimmune disease [17], we were unable to detect a bias toward a particular T_FH_ subset in patients with RA (Figure 2). As significant alterations in T cell profiles can occur following density-gradient purification (Supplementary Figure 3) [47], these effects may influence T_FH_ cell characterization, and further studies comparing T_FH_ subsets in whole blood versus purified PBMC will be important.

In conclusion, results presented here provide evidence that T_FH_ cells have a role in the pathogenesis of RA and suggest that qualitative differences in the expression of inhibitory receptors may be important to the immune response and efficacy of treatment with anti-TNF agents. The upregulation of CD200 on T_FH_ cells in RA patients with autoantibodies and those receiving treatment with anti-TNF*α* therapies supports a causal link between inflammation and induction of this receptor. Future studies examining CD200 and its ligand will contribute further to our understanding of the pathogenesis of RA and may help to identify new therapeutic targets in this disease.

## Supplementary Material

Supplementary Figure 1: Expression of stimulatory receptors on T_FH_ cells in RA Expression of CD134 (OX-40), CD69, HLA-DR and CD95 in patients with RA compared with controls. There were no significant differences between any of the groups (p>0.05). Each spot represents an individual patient. Mean ± SEM is shown.
Supplementary Figure 2: Induction of CD200 on T_FH_ cells is not due to a circulating factor in patients with high CD200 expression. PBMC from healthy subjects were incubated with serum from RA patients with high or low levels of CD200 expression on T_FH_ cells, and CD200 levels assessed at 24 hours, 48 hours or 72 hours. CD200 expression on T_FH_ cells was induced by PHA but not by serum from patients with low or high CD200 expression (p>0.05). Black columns are unstimulated controls and white columns stimulated samples. Columns shown the mean ± SEM and circles the individual values.
Supplementary Figure 3: Increased CD200 expression *in vitro* following purification and culture. CD200 expression is significantly increased on freshly purified PBMCs-PBMC (F), when compared with whole blood (WB) (p=0.018) or PBMCs that have been rested for 24 hours before analysis-PBMC (R) (p=0.021). Each spot represents an individual subject. Mean ± SEM is shown.

## Figures and Tables

**Table 1:** Characteristics of the study population*.

Characteristic	Seropositive (*n* = 20)	Seronegative (*n* = 15)	Controls (*n* = 15)	*P* values
Age	59.4 (33.2–90.4)	60.2 (32.7–80.9)	56.3 (22.4–84.8)	0.87
% Male	50	46.7	53.3	0.94
Lymphocytes^†^	1.94 (0.84–3.26)	1.95 (0.67–3.18)	2.1 (1.0–3.24)	0.95
% CD4^+^	50.2 (26.8–66.9)	56.2 (37.6–68.3)	44.43 (28.5–65.4)	0.02
% CD8^+^	22.3 (8.21–59.1)	17.3 (4.53–51.4)	25.97 (11.2–39.6)	0.03
CRP (mg/L)	33.7 (2–132)	30.29 (2–101)	N/A	0.86
ESR (mm/hr)	24.8 (2–105)	22.2 (1–84)	N/A	0.75
Creatinine (*μ*mol/L)	80.4 (49.3–105.2)	82.8 (50.4–139.7)	N/A	0.95
DAS-28 (CRP)	4.20 (0.97–6.66)	4.74 (2.10–8.63)	N/A	0.84
Duration of RA	52.6 (1–232)	46.5 (1–135)	N/A	0.87
Steroid therapy	6/20	6/15	N/A	0.72
DMARD therapy	16/20	9/15	N/A	0.14
Anti-TNF therapy^‡^	9/20	6/15	N/A	0.77

*values shown are the mean and range (brackets).

^†^×10^6^/mL.

^‡^5 patients received etanercept (3 in the seropositive and 2 in the seronegative group); the remaining patients received adalimumab.
