# Supplementary figures and images for: The Phenotype of Circulating Follicular-Helper T Cells in Patients with Rheumatoid Arthritis Defines CD200 as a Potential Therapeutic Target
Source: Clin Dev Immunol. 2012 Oct 4;2012:948218. doi: 10.1155/2012/948218 (PMC3471455; doi:10.1155/2012/948218)

Supplementary Figure 1

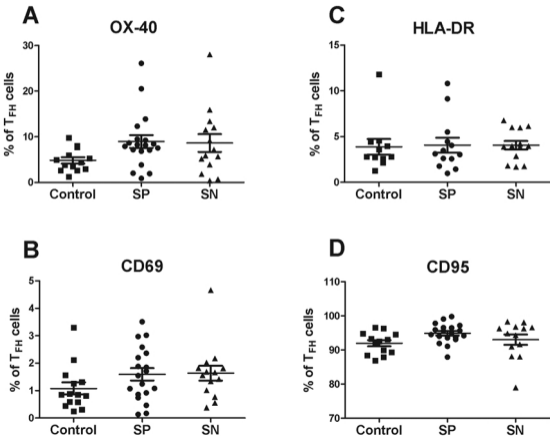

Supplement: Supplementary file 1 — Supplementary Figure 1: Expression of stimulatory receptors on TFH cells in RA Expression of CD134 (OX-40), CD69, HLA-DR and CD95 in patients with RA compared with controls. There were no significant differences between any of the groups (p>0.05). Each spot represents an individual patient. Mean ± SEM is shown. Supplementary Figure 2: Induction of CD200 on TFH cells is not due to a circulating factor in patients with high CD200 expression. PBMC from healthy subjects were incubated with serum from RA patients with high or low levels of CD200 expression on TFH cells, and CD200 levels assessed at 24 hours, 48 hours or 72 hours. CD200 expression on TFH cells was induced by PHA but not by serum from patients with low or high CD200 expression (p>0.05). Black columns are unstimulated controls and white columns stimulated samples. Columns shown the mean ± SEM and circles the individual values. Supplementary Figure 3: Increased CD200 expression in vitro following purification and culture. CD200 expression is significantly increased on freshly purified PBMCs-PBMC (F), when compared with whole blood (WB) (p=0.018) or PBMCs that have been rested for 24 hours before analysis-PBMC (R) (p=0.021). Each spot represents an individual subject. Mean ± SEM is shown. [file 948218.f1.pdf]

Supplementary Figure 2

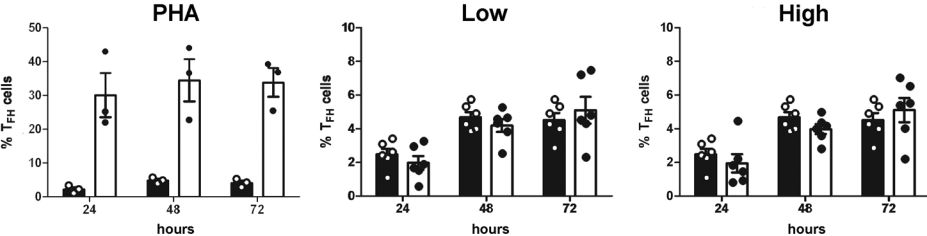

Supplement: Supplementary file 2 [file 948218.f2.pdf]

Supplementary Figure 3

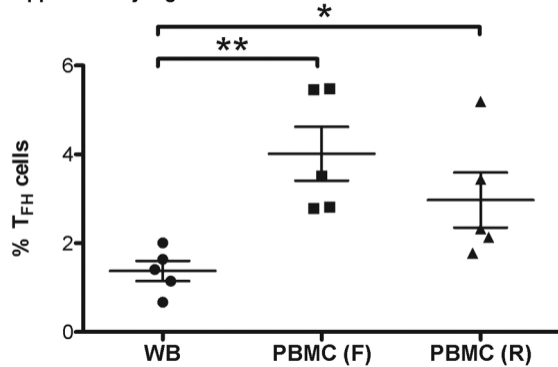

Supplement: Supplementary file 3 [file 948218.f3.pdf]
